# Immune System Remodelling by Prenatal Betamethasone: Effects on β-Cells and Type 1 Diabetes

**DOI:** 10.3389/fendo.2020.00540

**Published:** 2020-08-11

**Authors:** David Perna-Barrull, Anna Gieras, Silvia Rodriguez-Fernandez, Eva Tolosa, Marta Vives-Pi

**Affiliations:** ^1^Immunology Section, Germans Trias i Pujol Research Institute, Autonomous University of Barcelona, Badalona, Spain; ^2^Department of Immunology, University Medical Center Hamburg-Eppendorf, Hamburg, Germany

**Keywords:** prenatal betamethasone, Type 1 diabetes, immune system, β cell, glucocorticoid

## Abstract

Type 1 diabetes (T1D) is a multifactorial disease of unknown aetiology. Studies focusing on environment-related prenatal changes, which might have an influence on the development of T1D, are still missing. Drugs, such as betamethasone, are used during this critical period without exploring possible effects later in life. Betamethasone can interact with the development and function of the two main players in T1D, the immune system and the pancreatic β-cells. Short-term or persistent changes in any of these two players may influence the initiation of the autoimmune reaction against β-cells. In this review, we focus on the ability of betamethasone to induce alterations in the immune system, impairing the recognition of autoantigens. At the same time, betamethasone affects β-cell gene expression and apoptosis rate, reducing the danger signals that will attract unwanted attention from the immune system. These effects may synergise to hinder the autoimmune attack. In this review, we compile scattered evidence to provide a better understanding of the basic relationship between betamethasone and T1D, laying the foundation for future studies on human cohorts that will help to fully grasp the role of betamethasone in the development of T1D.

## Betamethasone as an Emerging Environmental Factor in T1D

Type 1 diabetes (T1D) is an autoimmune disease caused by the selective destruction of insulin-producing β-cells. The trigger, however, remains unknown. Postnatal environmental determinants have been thoroughly studied as risk factors (1, 2) but a crucial phase for the immune system development, the late prenatal stage, has been poorly investigated. Specifically, the interaction of drugs commonly used during late pregnancy with T1D and the pancreatic β-cells remains unexplored. Nonetheless, some studies reveal the importance of the prenatal stage and the prematurity of the newborn in the development of T1D (3–5). An indirect demonstration of how critical the *in utero* environment is in T1D development arises from the studies in twins: heterozygotic twins have an increased concordance of T1D when compared to non-twin siblings (6, 7), underlining the potential relevance of prenatal factors and their influence in the development of autoimmunity.

Synthetic glucocorticoids, most often betamethasone, are routinely given to mothers at risk of preterm birth between 24 and 34 weeks of gestation. A single course of prenatal betamethasone reduces the occurrence and severity of respiratory distress syndrome and improves the survival chances in premature infants (8, 9). Another glucocorticoid used for lung maturation is dexamethasone and produces similar results on the newborn survivability (10). These synthetic glucocorticoids cross the placenta and accelerate foetal lung maturation, achieving maximum benefit between 24 h and 7 days after administration (11). Betamethasone is a poor substrate for the glucocorticoid inactivating enzyme 11beta-hydroxysteroid-dehydrogenase 2 (11βHSD2), therefore, its bioactivity in the foetus lasts for several days (12) and it is known to exert long-lasting effects on the hypothalamic-pituitary-adrenal (HPA) axis and cognition in children (13, 14).

Glucocorticoids exert their effects by binding nuclear receptors that are ligand-dependent transcription factors. They can regulate gene transcription, either by direct binding to DNA or by interacting with other transcription factors (15). Glucocorticoid receptors (GR) are ubiquitously expressed; however, due to the variation in the genomic location of GR binding, the transcriptional responses to glucocorticoids are cell type-specific (16). Moreover, polymorphisms of the GR result in alterations in their responsiveness to glucocorticoids and in gene expression (17, 18). In addition, human GR receptor can be a target of endoncrine disruptors such as pesticides (19) that, in combination with antenatal glucocorticoids, could increase developmental neurotoxicity (20).

The general effects of glucocorticoids administered during pregnancy have been thoroughly reviewed (21). Considering the overwhelming use of betamethasone as the treatment of choice for respiratory distress syndrome in premature infants and the cell-specific response to glucocorticoids, in this review we will dissect the specific effects of betamethasone on the main cellular players in the context of T1D, namely immune cells and their targets, the β-cells of the pancreas.

## Direct Effects of Betamethasone on the Immune System

Several cell types of the immune system are involved in the development of T1D, and disturbances in the activity of these cells, such as enhanced proinflammatory activity, can increase the risk to develop T1D (22). Below, the effect of betamethasone on different cell types of the immune system is detailed.

### Innate Immune Cells

Prenatal administration of betamethasone can induce an anti-inflammatory status in the newborn during the first days after delivery (23), and this fact could be due to the immunomodulatory effects of betamethasone on innate immune cells.

### Neutrophils

Neutrophils have gained interest in T1D aetiology due to their participation in the initial steps of autoimmunity against β-cells (24). Moreover, neutrophils are part of the islet leukocytic infiltrates of patients with T1D, and are accordingly reduced in peripheral blood at disease onset (25, 26).

A described effect of betamethasone is the increase in leukocyte counts in peripheral blood after treatment (27), similarly to the effects of natural glucocorticoids during stress (28). Accordingly, neutrophil number and percentage were increased in human blood after betamethasone treatment (29), correlating with the described neutrophil demargination into the blood vessels (30–32). Moreover, in humans, betamethasone reduces neutrophil motility and chemotaxis (33), and can affect metabolism and cytokine production, i.e., reducing interleukin (IL)-8 and macrophage inflammatory protein alpha (MIP-1α) release (34). The inflammatory capacity of neutrophils is therefore reduced, as demonstrated in a lamb model of lung inflammation after betamethasone treatment, where gene expression of *IL-1, IL-6, IL-8*, and *CCL2* was suppressed (35).

### Monocytes

Monocytes are circulating innate immune cells that can become antigen-presenting cells (APCs), either macrophages, or dendritic cells (DCs). Thus, reprogramming monocytes may lead to changes in both differentiated cells. Betamethasone has an acute effect on the metabolism of monocytes, transiently reducing the production, and the secretion of IL-6 and reactive oxygen species. By contrast, the phagocytic activity of monocyte-derived APCs was not altered by betamethasone (36). In newborn children with low weight at birth, prenatal betamethasone administration induced a transient immunomodulatory effect in monocytes, causing diminished IL-6 and IL-10 release and downregulation of human leukocyte antigen DR (HLA-DR) expression (37). Moreover, the total number of monocytes was reduced by betamethasone (38). This effect was also assessed *in vitro*, demonstrating that glucocorticoids induce apoptosis in human monocytes (39). Nevertheless, these results are controversial, and other authors reported that betamethasone does not affect monocytes' IL-6 production (40).

### Macrophages

Macrophages are crucial in the initial damage to β-cells in T1D. These tissue-resident APCs contribute to initiate specific immune responses (41). In macrophages, betamethasone diminishes cytokine secretion (IL-8 and TNFα) (42) and impairs their ability for antigen presentation to T cells (43). These effects point to the induction of a regulatory profile in macrophages, similar as described in M2 macrophages (44). Indeed, dexamethasone induces the polarization of the M2 phenotype (45). Moreover, it was recently reported that dexamethasone increases the migration of macrophages by CD26 overexpression, a membrane glycoprotein with enzymatic capabilities involved in inflammation, and this could contribute to the egress of macrophages from inflamed tissue (46).

### Dendritic Cells

DCs are professional APCs with the ability to stimulate naïve T cells. In T1D, DCs are responsible for the presentation of β-cell autoantigens to T lymphocytes, initiating the adaptive autoimmune response against the insulin-producing cells. Similarly to the observed effect of dexamethasone on this cell type, DCs differentiated *in vitro* in the presence of betamethasone failed to achieve a fully mature status, showing a reduced capacity to stimulate the production of IL-17, a cytokine involved in autoimmune responses, by T lymphocytes (32). Furthermore, the release of proinflammatory cytokines was reduced by this drug in DCs. Betamethasone has been reported to induce tolerogenic Langerhans DCs (LDCs) in the skin of patients with psoriasis (47) and atopic dermatitis, which in turn arrest T helper (Th) 1 and Th2 responses (48). Similarly to the effects found in monocytes, human DCs differentiated with betamethasone showed a reduction of membrane expression of costimulatory molecules, such as CD40 and CD86, accompanied by a decrease in IL-12 secretion, an important cytokine for Th1 responses. These effects resulted in tolerogenic function in DCs and impaired ability to induce T lymphocyte proliferation (39).

### Natural Killer Cells

Natural Killer cells (NKc) are effector lymphocytes of the innate immune system. Their role in T1D is not completely understood, but abnormalities in this cell type may contribute to trigger autoimmune reactions against β-cells (49). Little is known about the effects of betamethasone on NKc. Betamethasone tends to increase NKc activity in very preterm newborn babies (<32 weeks of gestation), supporting the maturation of this cell type (40). However, other studies have reported that betamethasone reduces the number of NKc in newborn infants (50). In adults, NKc showed a reduced cytolytic activity after topical betamethasone administration (51).

### Adaptative Immune Cells

Innate immune cells are crucial in the first phases of autoimmune diseases, but the final effector cells are the adaptative immune system cells, T and B lymphocytes. Modulation of these cells can dampen or exacerbate an autoimmune reaction.

### T Lymphocytes

Betamethasone treatment results in T cell precursor apoptosis and, to a lesser extent, of mature T cells. Transient reduction in thymus weight and thymocyte numbers have been described after prenatal betamethasone administration in mice (32, 52). In humans, the thymus of the foetus of mothers that were prenatally treated with steroids showed delayed growth (53). Moreover, a reduction of 20–30% of peripheral lymphocyte counts was observed in pregnant women after treatment with betamethasone, although this effect only lasted for 3 days (27, 38, 54). Other glucocorticoids, like dexamethasone, have comparable effects on lymphocytes after prenatal treatment (55). In newborn children, a similar effect on lymphocyte counts has been described, mainly affecting CD4+ T lymphocytes (56). Data on lymphocyte counts are still controversial, since a different study reported an increase in CD3+ T lymphocytes in very preterm newborn babies (<32 gestational weeks) after betamethasone treatment (50). Prenatal betamethasone administered to the experimental model of T1D, the non-obese diabetic (NOD) mouse, resulted in long-lasting changes in the T Cell Receptor (TCR) Vβ repertoire that persisted into adulthood (32). Importantly, the TCR Vβ families that diminished in frequency after prenatal steroid treatment included pathogenic Vβ domains (57, 58), so it is reasonable to speculate that betamethasone will protect against T1D. In humans, betamethasone reduces T lymphocyte proliferation capacity (40), thus reducing clonal expansion. Overall, T cells will have an impaired capacity of interacting with β-cell antigens, thus contributing to prevent the autoimmune response.

### B Lymphocytes

The role of B lymphocytes in the development of T1D is not completely understood. B cells produce autoantibodies to islet antigens that, even if extremely useful as predictive biomarkers for disease, do not appear pathogenic. Also, B cells are critical as APCs during the first stages of autoimmunity in T1D (59). Betamethasone has a deleterious effect on mature B cells of NOD mouse (32) and reduces their ability to produce antibodies, specifically IgE and IgG (60, 61). Other glucocorticoids, such as dexamethasone, show a similar deleterious effect (62), affecting early precursor B cells, whereas mature B cells –IgD positive– are resistant to glucocorticoid-induced apoptosis (63). The reduction observed in antibody production could be the result of impaired B cell receptor and Toll-like receptor 7 signalling, since without these signals B cells cannot switch their Ig isotype, thus reducing their functionality. At the same time, B cells have increased transcriptional activity of *IL-10*, amplifying the immunomodulatory capacity of these cells induced by glucocorticoids (16).

## Effects of Betamethasone on the Target Cells of Autoimmune Diabetes, the Islet β-Cells

β-cells are the insulin-producing cells of the islets of Langerhans. The autoimmune destruction of these cells is the ultimate cause of T1D. β-cells also have an active role in their own destruction, facilitating the interaction with the immune system, and contributing to their own demise (64). Thus, the identification of changes induced by betamethasone may help to understand the possible outcome of this drug in the context of T1D. Studies performed in subjects with long term glucocorticoid treatment indicate that glucocorticoids can induce dysglycaemia, leading to diabetes (65, 66). Glucocorticoids increase insulin resistance (67) without affecting β-cell mass (68). On the other hand, physiological endogenous levels of glucocorticoids are necessary for maintaining the regulation of insulin secretion by β-cells (69). Moreover, prenatal glucocorticoids support the maturation of β-cells by enhancing their glucose sensitivity due to increased expression of *Glut2* and *Gck* genes and by reducing apoptosis, similarly as the overexpression of surfactant proteins induced by glucocorticoids helps with the maturation of the foetal lungs (70). In a similar way, prenatal glucocorticoids enhance insulin secretion in rats due to the overexpression of *Gck, Slca2*, and *Ins2* genes in β-cells, despite β-cell mass is smaller than in non-treated animals (71). Experimental data demonstrate that prenatal betamethasone reduces the risk of developing T1D in the NOD mice (57), correlating to altered expression of genes related to metabolism and autoimmunity in β-cells. In a previous study we reported a reduction of *Ccl2* gene expression in β-cells, which may lead to a reduced recruitment of macrophages and monocytes. Moreover, an increase in *Gad1* gene expression, could promote tolerance to β-cells in NOD mice (32). Recent studies in other T1D experimental models indicate that short-term treatment with betamethasone during late pregnancy does not affect β-cell metabolism in later life (72, 73). Glucocorticoid signalling can also cause epigenetic modifications in these cells. In fact, glucocorticoids impair the methylation of the DNA by altering the enzymes responsible for this process. Moreover, the prenatal period is a very sensitive phase during which the epigenome shows heightened plasticity to methylation modifications and these changes can be accumulated throughout life (74). Important β-cell functions, such as insulin secretion and islet cell mass homeostasis, are controlled by epigenetic mechanisms (75), and glucocorticoids can modify the epigenome of β-cells inducing changes that can affect their function in adults (76), altering the efficiency of glucose metabolism (77, 78).

## How can Betamethasone Affect the Interplay Between the Immune System and β-Cells?

**Figure 1:**
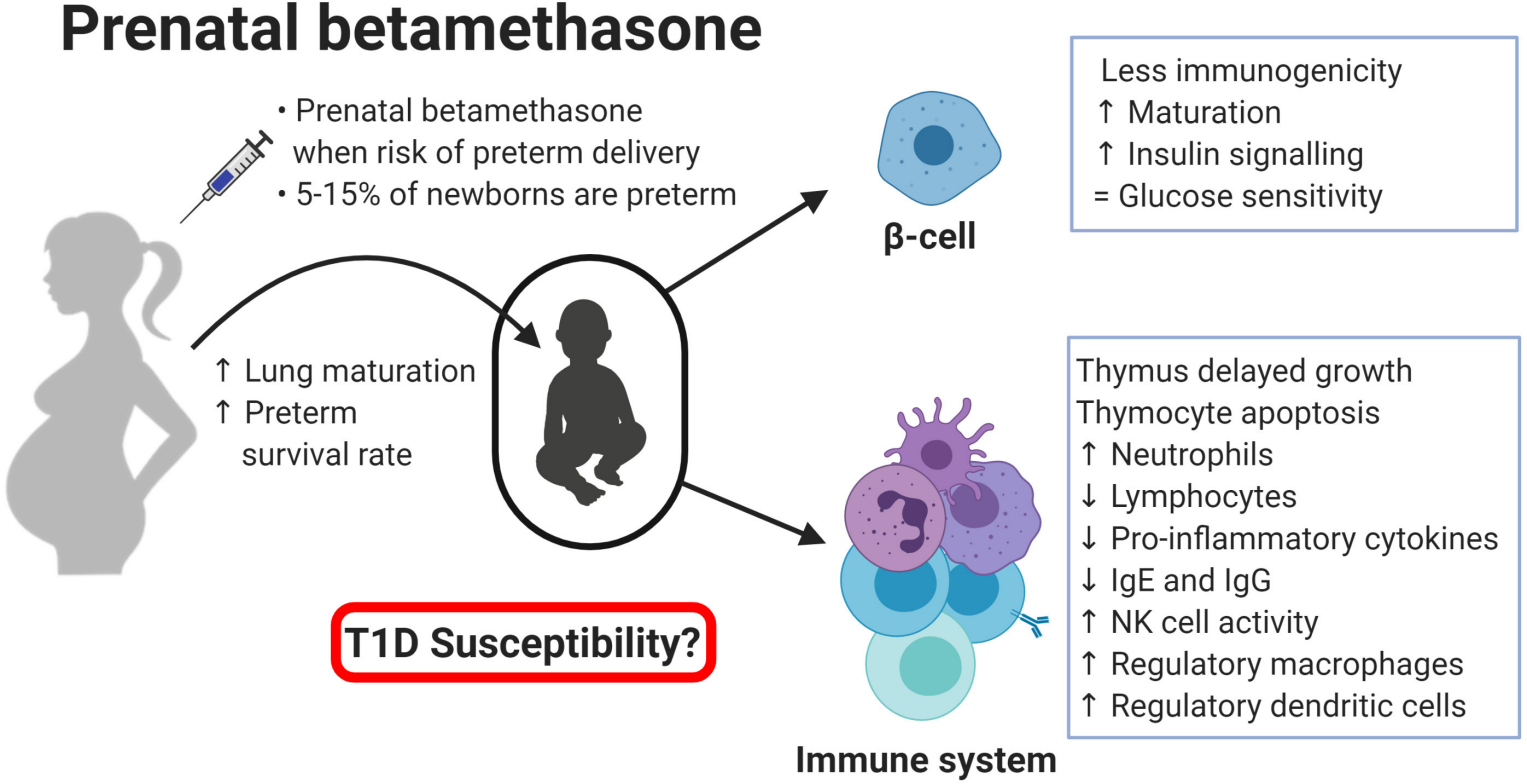
Effects of prental betamethasone on β-cells and the immune system of the newborn. Created with BioRender.com.

T1D is a multifactorial disease with complex interactions between the immune system and the pancreatic β-cells. Glucocorticoids are potent immune suppressors and are commonly used in patients with autoimmune diseases such as psoriasis or rheumatoid arthritis (79, 80). Betamethasone, like other synthetic glucocorticoids, can reduce cytokine production and release, thereby inhibiting specific immune responses and blocking the initiation of an autoimmune attack to β-cells (2). Dampening the autoimmune reaction can be the most efficient form of preventing T1D, and it might be a consequence of the impaired functionality of innate and adaptative immune cells (Figure 1). On the one side, betamethasone diminishes the proinflammatory action of innate immune cells (neutrophils, macrophages, and NK cells). On the other side, this drug induces a tolerogenic antigen presentation in macrophages and DCs, limiting the possibilities to activate autoreactive T lymphocytes in the lymph nodes. In turn, lymphocytes are also affected by betamethasone, as aforementioned. T lymphocytes reduce their proliferation capacity, decreasing the number of cells that can kill the β-cells. At the same time, prenatal treatment critically reduces the number of developing thymocytes and induces a skewed TCR repertoire towards T cells with less affinity to β-cell autoantigens (57). This fact will impair autoreactivity when APCs expose β-cell autoantigens in Major Histocompatibility Complex (MHC) molecules. Moreover, in the presence of betamethasone, T lymphocytes tend to differentiate to Th2 rather than autoimmunity-prone Th1 or Th17 cells (81), and it has been demonstrated that glucocorticoids exposure during foetal development can alter the HPA axis, impairing CD8+ T lymphocytes function later in life, making them less responsive against viral antigens (82), or blunting cortisol response against rhinovirus (83). In this sense, concerns have been raised about multiple doses of prenatal betamethasone, including an increased susceptibility to infections in children (84, 85). B cell precursors are also affected by betamethasone, showing reduced antibody production (63). However, how these effects altogether might influence the development of T1D is not yet known. Taken together, these alterations suggest a rather positive effect leading to T1D protection, but this effect could depend, among other factors, on the concentration, and duration of the prenatal treatment. In addition, β-cell changes induced by betamethasone may enhance this protective effect. Especially, β-cell maturation and the acquisition of an apoptosis-resistant phenotype may be key factors in thwarting undesired autoimmune reactions (70). Moreover, alterations found in the expression of genes related to interactions between the immune system and β-cell reduce β-cell immunogenicity, hindering their direct interaction with immune system cells (32). This could help to avoid the activation of stray cytotoxic T cells with affinity to β-cell autoantigens. How long this effect is maintained is unknown, but it is reasonable to speculate that prenatal betamethasone could reprogramme some aspects of β-cell function until adult life, without affecting their intrinsic capacities as described for insulin secretion (86). Furthermore, glucocorticoid stimulation also induces epigenetic changes in the precursor β-cells (76), and this could be the main actor behind the long-term effects observed after prenatal administration of betamethasone. We have summarised those studies that used betamethasone in their research (Table 1), focusing on their findings in the context of the immune system and β-cells, and the expected effect these changes would have on T1D. Further studies are needed to reveal the long-term effects of prenatal betamethasone treatment in the immune system and T1D (87).

**Table 1:** Studies on the effect of betamethasone in the immune system and impact on T1D.

**Species/substrates**	**Main findings**	**Expected effect on T1D**	**References**
Human	↓ NKc cytolytic capacity	Protective	(49)
Human	↓ IgE synthesis by B cells	Neutral	(59)
Human	↑ Insulin resistance (long-term) No effect in T1D prevalence (long-term)	Neutral	(80)
Human	↑ T1D Hazard ratio after glucocorticoid treatment	↑ Risk	(81)
Human	↑ Neutrophils ↓ Basophils, CD3+CD4+, and CD3+CD8+ T cells	Protective	(27)
Human (adult monocytes)	↓ DCs costimulatory molecules ↓ IL-12p70 ↓ Th1 activation	Protective	(37)
Human (adult skin cells)	↓ LDCs costimulatory molecules and HLA-DR ↓ Proinflammatory cytokines No effects in IL-10 secretion or ILT3 expression	Protective	(45)
Human (cord blood of preterm babies)	↑ NKc activity (<32 weeks gestation) ↓ Lymphocyte proliferation No effect in IL-6 secretion	Protective	(38)
Human (cord blood)	↓ IL-6, IL-8 and TNFα secretion by macrophages	Protective	(40)
Human (newborn and adult)	↓ migration and motility of newborn's neutrophils No effects in adult's neutrophils	Neutral	(31)
Human (newborn)	↓ IL-8 and CCL3 secretion from neutrophils	Protective	(33)
Human (newborn)	↓ HLA-DR expression on monocytes ↓ IL-6 and IL-10 in plasma	Protective	(35)
Human (newborn)	↑ CD3+ T cells and monocytes ↓ NKc	↑ Risk	(48)
Human (newborn)	↓ CD4^+^ and CD25^+^ T lymphocytes	Protective	(54)
Human (pregnant women)	↑ Leukocytes and granulocytes ↓ Lymphocytes	Neutral	(25)
Human (pregnant women)	↑ Leukocytes ↓ Lymphocytes and monocytes	Neutral	(36)
Human (pregnant women)	↑ Neutrophils ↓ Lymphocytes	Neutral	(52)
Mouse (NOD)	↓ Immunogenicity ↑ Tolerance	Protective	(30)
Mouse (NOD)	↓ T1D incidence ↓ Diabetogenic Vβ TCR	Protective	(55)
Mouse	↓ Impaired antigen presentation by macrophages	Protective	(41)
Mouse	↓ Th1 and Th2 induction by LDCs	Protective	(46)
Mouse	↑ Apoptosis of thymocytes ↓ Thymus weight	↑ Risk	(50)
Sheep	↓ IL-1, IL-6, IL-8, CCL2, and TLR4 expression	Protective	(33)
Sheep	↓ IL-6 and ROS from monocytes	Protective	(34)
Sheep	No long-term effects Improves preterm delivery adverse effects	Neutral	(69)
Sheep	No impairment of insulin sensitivity ↑ Insulin signalling pathway	Neutral	(71)
Rabbit	↓ B cells IgG+	Neutral	(58)

*CCL, C-C motif ligand; HLA, human leukocyte antigen; ILT3, immunoglobulin-like transcript 3; IL, interleukin; IgE, immunoglobulin E; IgG, immunoglobulin G; LDCs, Langerhans dendritic cells of the skin; NKc, Natural killer cells; ROS, reactive oxygen species; T1D; type 1 diabetes; TCR, T cell receptor; Th, T helper; TLR, toll like receptor; TNF, tumour necrosis factor*.

## Future Perspectives

The effectiveness of glucocorticoids has been demonstrated for a wide range of immunologically related diseases. However, their effects during the prenatal period, both in the immune system and the target tissue of T1D, are not fully characterised. Expanding the understanding of how they can affect self-tolerance and T1D could contribute to reduce the increasing incidence of this and other autoimmune diseases. Another key point is to determine whether the effects of glucocorticoid treatment found in immune system cells could result from changes induced in hematopoietic stem cells, thus explaining the alterations found in many immune cell types. Further studies are required to dissect the exact mechanism and the magnitude of these changes in the immune system and β-cells, because other factors, such as maternal nutrition or stress during pregnancy, could also veil betamethasone effects (88). Finally, epidemiological studies are needed to explore the effect of prenatal betamethasone on T1D. Finding pieces of evidence of the precise effects of betamethasone in T1D development could lead to improved neonatal care, with special focus on children with higher genetic risk to suffer from T1D.

## Conclusion

Knowledge about betamethasone action on the immune system is currently increasing, but it is very limited in the prenatal stage as well as in its consequences in the adulthood phase. Glucocorticoids have shown a plethora of effects, which depend on the duration of the treatment, the route of administration, the target tissue, etc. The key message of this review is that prenatal betamethasone affects the immune system cells, and these alterations may have long-term consequences. Simultaneously, betamethasone also alters β-cells towards a less immunogenic phenotype and could induce epigenetic modifications in immune cells and β-cell precursors. Considering these effects, it is tempting to speculate that betamethasone may act as a protective agent against human T1D when administered shortly before birth or in the perinatal period. Future immunological, metabolic, and epidemiological studies, together with the extrapolation of data from other glucocorticoids like dexamethasone, will shed light on the unanswered questions related to this prenatal treatment.

## Author Contributions

DP-B and MV-P wrote the manuscript. AG, SR-F, and ET participated in the literature review, and edited the manuscript while adding additional insights. All authors read and approved the final version of the manuscript.

## Conflict of Interest

MV-P holds a patent that relate to immunotherapy for T1D and is co-founder of Ahead Therapeutics S.L. SR-F is part-time employed at Ahead Therapeutics S.L. The authors have no other relevant affiliations or financial involvement with any organization or entity with a financial interest in or financial conflict with the subject matter or materials discussed in the manuscript apart from those disclosed.

The remaining authors declare that the research was conducted in the absence of any commercial or financial relationships that could be construed as a potential conflict of interest.
